# Artificial Intelligence in Digestive Endoscopy Training—The Past, Present, and Future

**DOI:** 10.1111/den.70047

**Published:** 2025-10-26

**Authors:** Jacky C. L. Ho, Zhouyao Qian, Louis H. S. Lau, Hon‐Chi Yip, Philip W. Y. Chiu

**Affiliations:** ^1^ Department of Medicine and Therapeutics, Faculty of Medicine The Chinese University of Hong Kong Hong Kong; ^2^ Li Ka Shing Institute of Health Sciences The Chinese University of Hong Kong Hong Kong; ^3^ Institute of Digestive Disease The Chinese University of Hong Kong Hong Kong; ^4^ Department of Surgery, Faculty of Medicine The Chinese University of Hong Kong Hong Kong

**Keywords:** artificial intelligence, CADe, CADx, capsule, education, endoscopy, hepatobiliary, luminal, therapeutic, training

## Abstract

**Background and Objective:**

Artificial intelligence (AI) is reshaping gastrointestinal endoscopy, yet its role in training remains unexplored. This narrative review summarizes current evidence on AI‐assisted endoscopy training, addresses potential drawbacks, and envisions future directions.

**Methods:**

This narrative review was performed via a systematic MEDLINE search (including articles from inception to January 2025), with search terms covering ‘AI’, ‘endoscopy,’ and ‘training.’ Studies were excluded if they were reviews, letters, editorials or comments; focused solely on model development; lacked a training component; or were limited to simple comparisons between the performance of endoscopists and AI systems. After screening 1443 records, 27 articles were included in this review.

**Results:**

AI demonstrates potential in enhancing the training of various types of endoscopy (including luminal, hepatobiliary, capsule, and therapeutic endoscopy) by improving quality metrics, enhancing lesion detection, and guiding anatomical landmark recognition, yet the current applications are mainly task‐based. Future AI must evolve to provide comprehensive training and personalized performance tracking to endoscopists of different levels of experience. Further studies are needed to assess objective educational outcomes and cost‐effectiveness. Key concerns for AI adoption, including deskilling, over‐reliance, ethical considerations, and practicality, should be addressed through structured implementation, quality assurance, and regulatory framework.

**Conclusion:**

In conclusion, AI can augment endoscopy training by improving skill acquisition and procedural quality, yet significant gaps remain. More research is needed to support its widespread integration.

## Graphical abstract

AI may enhance endoscopy training through improved detection, guidance and evaluation, but demands careful implementation, validation and ethical governance to maximize benefits.

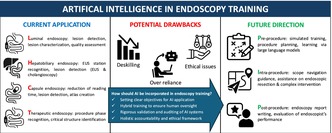

## 1 Introduction

### 1.1 The Past—History of AI Development

The concept of artificial intelligence (AI) was first described in the 1950s to reflect the use of computers to simulate human intelligence for multiple tasks. Over the past decades, the field of AI has gradually evolved from machine learning to deep learning and convolutional neural networks, which enabled the analysis of complex images and videos and led to diverse applications in medicine. In gastrointestinal (GI) endoscopy, AI application started with colonic polyps detection by computer vision, followed by different innovations in upper GI and hepatobiliary endoscopy, capsule endoscopy, and advanced therapeutics [1, 2].

### 1.2 Endoscopists' Perception

According to previous studies, although most endoscopists were positive toward AI in endoscopy, their acceptance varied based on individual experience and specific scenarios. A survey from the United States revealed that most endoscopists expressed strong interest in AI‐assisted technologies, particularly computer‐aided polyp detection (CADe). Over half of endoscopists favored a “diagnose‐and‐leave” strategy utilizing computer‐aided characterization (CADx) for polyp classification. Yet, concerns were raised regarding potential drawbacks, including higher costs, operator dependency, and prolonged procedure duration [3]. Similarly, a survey conducted in the Asia‐Pacific region showed that endoscopists demonstrated high acceptance and trust in AI‐assisted colonoscopy for colorectal polyp management, with the trust level highest for CADe, followed by CADx and computer‐aided intervention (CADi). The relationship between risk perception, acceptance, and trust in AI adoption within gastroenterology practice was complex. Surprisingly, less experienced endoscopists expressed stronger risk perception than experienced endoscopists [4]. While attitudes toward AI had little direct impact on acceptance, they indirectly shaped behavior through trust and beliefs, which should be prioritized in the process of AI education and training [5].

## 2 Objective

The impact of AI on endoscopy training is not yet fully understood, and integrating AI into training curricula faces numerous hurdles. This narrative review aims to summarize current evidence on AI‐assisted endoscopy training and explore key aspects such as the efficacy of AI‐driven education, potential drawbacks of AI adoption, and future directions for implementation.

## 3 Methods

### 3.1 Search Algorithm

**FIGURE 1:**
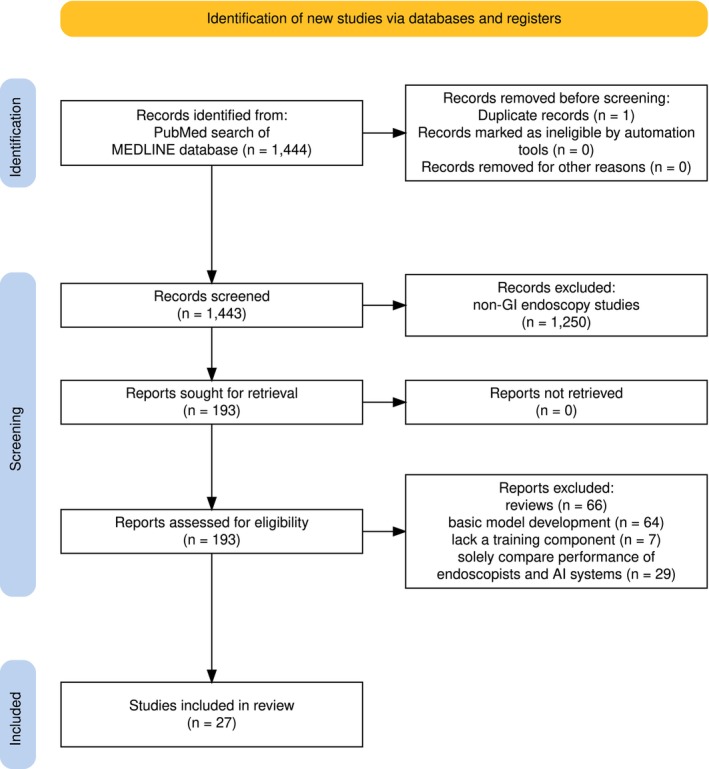
PRISMA flow diagram illustrates the study selection process for systematic review.

This narrative review was performed by a systematic search via the MEDLINE database. Articles written in English published from inception to January 31, 2025 were included. The complete search details were listed in Table 1. Briefly, the search strategy combined terms for AI (“artificial intelligence” or “deep learning” or “machine learning” or “neural network” or “computer‐aided” or “computer‐assisted”), training (“training” or “education”), and endoscopy (“luminal endoscopy” or “capsule endoscopy” or “hepatobiliary endoscopy” or “therapeutic endoscopy”). The selection process of articles was summarized in Figure 1. After the removal of duplicated records, the initial search yielded 1443 publications, of which 1250 non‐GI endoscopy studies were excluded. The remaining 193 full‐text articles were manually screened for relevance by the two authors (JCLH and ZQ) independently to minimize bias. Discordant selections were resolved through discussion. Studies were included if they featured an educational or training component and explicitly aimed to explore the application of AI in GI endoscopy training. Studies were excluded if they were reviews, letters, editorials or comments; focused solely on basic model development; lacked a training component; or were limited to simple comparisons between the performance of endoscopists and AI systems. As a result, 27 studies (11 RCTs and 16 prospective studies—8 in luminal endoscopy, 9 in hepatobiliary endoscopy, 5 in capsule endoscopy, and 5 in advanced therapeutics) were included in this narrative review (Table 2).

**TABLE 1:** Search strategy.

Strategy component	Query
Artificial intelligence #1	“artificial intelligence”[Title/Abstract] OR “deep learning”[Title/Abstract] OR “machine learning”[Title/Abstract] OR “neural network”[Title/Abstract] OR “computer‐aided”[Title/Abstract] OR “computer‐assisted”[Title/Abstract]
Training #2	“training”[All Fields] OR “education”[All Fields]
Luminal endoscopy #3	“luminal endoscopy”[Title/Abstract] OR “esophagogastroduodenoscopy”[Title/Abstract] OR “EGD”[Title/Abstract] OR “OGD”[Title/Abstract] OR “colonoscopy”[Title/Abstract]
Capsule endoscopy #4	“capsule endoscopy”[Title/Abstract] OR “wireless capsule endoscopy”[Title/Abstract] OR “video capsule endoscopy”[Title/Abstract]
Hepatobiliary endoscopy #5	“biliopancreatic endoscopy”[Title/Abstract] OR “endoscopic ultrasonography”[Title/Abstract] OR “endoscopic ultrasound”[Title/Abstract] OR “EUS”[Title/Abstract] OR “endoscopic retrograde cholangiopancreatoscopy”[Title/Abstract] OR “ERCP”[Title/Abstract] OR “cholangioscopy”[Title/Abstract]
Therapeutic endoscopy #6	“therapeutic endoscopy”[Title/Abstract] OR “endoscopic mucosal resection”[Title/Abstract] OR “EMR”[Title/Abstract] OR “endoscopic submucosal dissection”[Title/Abstract] OR “ESD”[Title/Abstract] OR “natural orifice transluminal endoscopic surgery”[Title/Abstract] OR “NOTES”[Title/Abstract] OR “endoscopic full thickness resection”[Title/Abstract] OR “EFTR”[Title/Abstract] OR “peroral endoscopic myotomy”[Title/Abstract] OR “POEM”[Title/Abstract] OR “endoscopic variceal ligation”[Title/Abstract] OR “EVL”[Title/Abstract] OR “endoscopic resection”[Title/Abstract] OR “submucosal dissection”[Title/Abstract] OR “endoscopic hemostasis”[Title/Abstract] OR “endoscopic ampullectomy”[Title/Abstract] OR “polypectomy”[Title/Abstract] OR “mucosectomy”[Title/Abstract]
Merge	
#7	#3 OR #4 OR #5 OR #6
#8	#1 AND #2 AND #7

**TABLE 2:** Summary of current applications of artificial intelligence in gastrointestinal endoscopy training.

Author, year	Country	Category	AI type	Study type	Study design	Endoscopist experience	Outcomes
Luminal endoscopy							
Repici, 2022 [6]	Italy, USA, Switzerland and Germany	Detection of polyps	CADe	Clinical study	RCT	Non‐expert (*N* = 10): < 2000 colonoscopies	Compare ADR of CADe group (*n* = 330) vs. control group (*n* = 330): 53.3% vs. 44.5% (*p* < 0.01)
Lau, 2024 [7]	Hong Kong	Detection of polyps	CADe (ENDO‐AID)	Clinical study	RCT	Endoscopist in training: < 500 procedures and < 3 years' experience (beginner [*N* = 12]: < 200 procedures; intermediate [*N* = 10]: 200–500 procedures)	Compare ADR of CADe group (*n* = 386) vs. control group (*n* = 380): 57.5% vs. 44.5% (*p* < 0.01)
Yamaguchi, 2024 [8]	Japan	Detection of polyps	CADe+CADx (CAD EYE)	Clinical study	RCT	Trainee (*N* = 6): third or fourth year as a physician + 0–20 colonoscopies Expert: > 5000 colonoscopies	Compare AMR of AI‐assisted group (*n* = 113) vs. control group (*n* = 118): 25.6% vs. 38.6 (*p* = 0.033)
Yao, 2024 [9]	China	Detection of polyps	CADe	Clinical study	RCT	Novice (*N* = 8): > 1 year experience in gastroenterology and no prior experience or training in colonoscopy Expert (*N* = 10): > 5000 colonoscopies	Compare AMR of AI‐assisted novice group (*n* = 227) vs. control novice group (*n* = 229): 18.82% vs. 43.69% (*p* < 0.001) Compare AMR of AI‐assisted novice group (*n* = 227) vs. control expert group (*n* = 229): 18.82% vs. 26.97% (*p* = 0.202)
Peng, 2024 [10]	China	Diagnosis of colorectal cancer	CADx	Pre‐clinical study	Prospective diagnostic study	Novice (*N* = 6): 200–400 colonoscopies Expert (*N* = 4): > 2000 colonoscopies	Develop an AI system and compare NPV of AI‐assisted group (*n* = 84) vs. non‐AI‐assisted group (*n* = 84) in novices: 88.2% vs. 50.0% (*p* = 0.013)
Weigt, 2022 [11]	Germany and Italy	Detection and characterization of colorectal neoplasia	CADe+CADx	Pre‐clinical study	Prospective diagnostic study	Non‐expert (*N* = 3): < 100 colonoscopies Expert (*N* = 3): > 10,000 colonoscopies	Develop an AI system and compare sensitivity and specificity of CADx‐assisted non‐expert group (*n* = 267) vs. CADx‐unassisted expert group (*n* = 267): WLI: sensitivity: 78.6% vs. 72.3% (*p* < 0.05); specificity: 75.4% vs. 82.5% (*p* < 0.05) BLI: sensitivity: 86.0% vs. 79.0% (*p* < 0.05); specificity: 62.7% vs. 80.4% (*p* < 0.05)
Rex, 2024 [12]	USA	Diagnosis of colorectal polyps	CADx	Clinical study	Prospective study	Not specified	Polyps measuring 5 mm or less from 1252 patients reviewed by 49 endoscopists (CADx assisted *n* = 2695; CADx‐unassisted *n* = 2695): sensitivity: 90.81% vs. 90.69% (*p* = 0.52); specificity: 64.66% vs. 59.54% (*p* < 0.001)
Zhang, 2023 [13]	China	Endoscopy reporting	Deep learning (AI‐EARS)	Clinical study	Prospective crossover study	Junior (*N* = 4): < 2 years of experience Senior (*N* = 4): 2–6 years of experience Expert (*N* = 4): > 10 years of experience	Develop an AI system and compare the completeness and accuracy of AI‐assisted group (*n* = 44) vs. AI‐unassisted group (*n* = 44): completeness of the textual description:79.03% vs. 51.86% (*p* < 0.001) accuracy of the textual description: 64.47% vs. 42.81% (*p* < 0.001) completeness of the photo documents of landmarks: 92.23% vs. 73.69% (*p* < 0.001) reporting time for an individual lesion: 80.13 vs. 46.47 s (*p* < 0.001)
Hepatobiliary endoscopy							
Robles‐Medranda, 2024 [14]	Ecuador and Romania	Recognition of normal anatomic structure	Deep learning (CNN)	Pre‐clinical study	Prospective diagnostic study	Expert (*N* = 2): > 250 EUS procedures per year	Develop an AI system and compare the performance of the AI model vs. experts through 25 videos: Sensitivity: 99.8% vs. 75.8%; Specificity: 100% vs. 98.7%; PPV: 100% vs. 82.6%; NPV:100% vs. 98.7%; Agreement: 100% vs. 98.7%
Zhang, 2020 [15]	China	Recognition of stations and monitoring of pancreatic vision loss	Deep learning (DCNN)	Pre‐clinical study	Prospective crossover study	Trainee (*N* = 8): 1 year of gastroenterology fellowship experience + no prior experience or training in EUS	Develop an AI system and compare the accuracy in station recognition with vs. without augmentation in trainees through 168 images: 67.2% vs. 78.4% (*p* < 0.01)
Yao, 2021 [16]	China	Annotation of bile duct and recognition of stations	Deep learning (DCNN)	Pre‐clinical study	Prospective crossover study	Primary trainees (*N* = 8): > 1 year gastroenterology experience + no prior experience or training in EUS Advanced trainees (*N* = 4): > 100 training EUS	Develop an AI system and compare the accuracy with vs. without augmentation in trainees through 29 videos: 76.3% vs. 60.8% (*p* < 0.01)
Tang 2023 [17]	China	Capture and segmentation of solid pancreatic masses	Deep learning (CH‐EUS MASTER)	Pre‐clinical study	Prospective crossover study	EUS trainees (*N* = 8): > 1 year of experience in endoscopy + 0.5 month of EUS learning	Develop an AI system and compare the performance before vs. after AI training in trainees through 30 videos and 60 images: IoU: 0.80 vs. 0.87 (*p* = 0.002) Time for identifying lesions in the pancreatic body/tail: 22.75 vs. 17.98 s (*p* < 0.001) Time for identifying lesions in the pancreatic head/uncinate: 34.21 vs. 25.92 s (*p* < 0.001)
Cui, 2024 [18]	China	Diagnosis of pancreatic solid lesions	Deep learning (CNN)	Pre‐clinical and clinical study	Prospective randomized crossover study	Novice (*N* = 6): > 1‐year experience Senior (*N* = 4): > 5‐year experience + ≥ 150 EUS per year Expert (*N* = 2): > 10‐year experience + ≥ 300 EUS per year	Develop an AI system and compare the accuracy of AI‐assisted novice group vs. AI‐unassisted novice group through 130 patients with EUS images and clinical data: 0.90 vs. 0.69 (*p* < 0.001)
Gu, 2023 [19]	China	Diagnosis of pancreatic ductal adenocarcinoma	Deep learning radiomics	Pre‐clinical study	Prospective study	Junior (*N* = 4): 2–4 years EUS experience Senior (*N* = 3): > 10 years EUS experience	Develop an AI system and the diagnostic accuracy of AI‐assisted junior group vs. AI‐unassisted senior group in 71 PDAC lesions and 52 benign lesions: higher than or comparable to
Li, 2024 [20]	China	Capture of standard stations, lesions, and puncture procedures	Deep learning (DCNN)	Pre‐clinical and clinical study	Prospective study	Junior (*N* = 2): < 5 years EUS experience Senior (*N* = 3): 5–10 years EUS experience Expert (*N* = 4): > 10 years EUS experience	Develop an AI system and compare completeness of AI system vs. endoscopists through 114 EUS procedures: 91.4% vs. 78.1% (*p* < 0.001)
Huang, 2021 [21]	China	Evaluation of stone size, CBD diameter, and ERCP technical difficulty	Deep learning (CasNet)	Pre‐clinical study	Retrospective study	Non‐expert (*N* = 2): > 3‐year ERCP experience + > 500 ERCP Expert (*N* = 6): > 10‐year ERCP experience + > 1000 ERCP	Develop an AI system and compare the consistence of technical difficulty scoring performance of AI system vs. nonexpert through 80 images: Kappa: 0.740 vs. 0.315 vs. 0.429
Robles‐Medranda, 2023 [22]	Ecuador, USA, Belgium and Spain	Detection of neoplastic lesions during DSOC	Deep learning (CNN)	Pre‐clinical study	Prospective study	Non‐expert (*N* = 4): general practitioner Expert (*N* = 4): > 150 DSOCs per year	Develop an AI system and compare the AUC of AI system vs. endoscopists through 170 patients: Non‐expert#2: CRM:0.794 vs. 0.657 (*p* < 0.05); Mendoza: 0.794 vs. 0.582 (*p* < 0.05) Non‐expert #4: CRM:0.791 vs. 0.683 (*p* < 0.05) Expert #4: CRM:0.848 vs. 0.755 (*p* < 0.05); Mendoza: 0.848 vs. 0.755 (*p* < 0.05)
Capsule endoscopy							
Postgate, 2009 [23]	UK	Educational effectiveness	Not specified	Pre‐clinical study	Prospective educational evaluative study	Novice (*N* = 14): no prior endoscopic or CE experience Trainee (*N* = 14): no prior CE experience with a median of 2250 gastroscopies and 2750 colonoscopies Expert (*N* = 4): ≥ 100 ce independently reported	Develop an AI system and compare the test performance of post‐AI training vs. pre‐AI training in trainees and novices through a 60‐queation test: Trainees:62.1% vs. 49.5% (*p* < 0.001) Novices: 46.7% vs. 29.5% (*p* < 0.001) Trainees+ Novices: 54.4% vs. 39.5% (*p* < 0.001)
Aoki, 2020 [24]	Japan	Reading time and detection of breaks (erosions or ulcerations)	Deep learning (CNN)	Pre‐clinical study	Retrospective study	Trainee (*N* = 4): < 10 CE review Expert (*N* = 2): > 400 CE review	Compare the reading time and detection rate of erosions or ulcerations of AI‐assisted vs. AI‐unassisted in trainees through reading 20 videos: reading time: 5.2 vs. 20.7 min (*p* < 0.001); detection rate of erosions or ulcerations: 55% vs. 47% (*p* > 0.05) Compare the reading time and detection rate of erosions or ulcerations of AI‐assisted trainee group (*n* = 4) vs. AI‐unassisted expert group (*n* = 2): reading time: 5.2 vs. 12.2 min (*p* < 0.001); detection rate of erosions or ulcerations: 55% vs. 84% (*p* = 0.003)
Xie, 2024 [25]	China	Reading time and detection of gastric and SB lesions	Deep learning (SS Plus)	Pre‐clinical study	Retrospective study	Junior (*N* = 3): 3 years of CE reading, > 60/year Senior (*N* = 3): 10 years of CE reading, > 200/year Adjudication (*N* = 3): 15 years of CE reading, > 300/year	Develop an AI system and compare the reading time, sensitivity and accuracy of AI‐assisted reading vs. AI‐unassisted reading in junior endoscopists through 342 ce examinations: Reading time: 3.05 vs. 98.81 min (*p* < 0.001) Sensitivity: 98.08% vs. 92.65% (*p* < 0.001) Accuracy: 98.25% vs. 93.27% (*p* < 0.001)
Ding, 2023 [26]	China	Diagnosis of multiple abnormalities	Deep learning (CNN + CRNN)	Pre‐clinical study	Retrospective study	Not specified	Develop an AI system and compare the overall accuracy of AI‐assisted junior endoscopists vs. AI‐unassisted junior endoscopists through 240 SBCE videos: 97.9% vs. 85.5% (*p* < 0.001, Bonferroni corrected) Compare the overall accuracy of AI‐assisted junior endoscopists vs. AI‐unassisted expert endoscopists through 240 SBCE videos: 97.9% vs. 96.6% (*p* > 0.0125, Bonferroni corrected)
Vats, 2023 [27]	Norway	Creation of a realistic WCE atlas	Generative Adverserial Networks (StyleGAN2)	Pre‐clinical study	Feasibility study	Experts (*N* = 8): 3–36 years' experience	Create an WCE atlas and validate its realism and plausibility in experts by 3 subjective 8 online experiments.
Therapeutic endoscopy							
Cao, 2023 [28]	Hong Kong, China, Singapore, and Germany	Phase recognition for ESD	Deep learning (AI‐Endo)	Pre‐clinical study	Retrospective study	Not specified	Develop an AI system for ESD phase recognition and validate it in diverse settings including ex vivo (*n* = 4), in vivo (*n* = 12) and human (*n* = 27) ESD procedures: overall accuracy range from 83.53% to 93.07%
Furube, 2024 [29]	Japan	Phase recognition for ESD	Deep neural network	Pre‐clinical study	Retrospective study	Not specified	Develop an AI system for ESD recognition and validate its performance: accuracy, precision, recall, and F value rates 90%, 91%, 90%, and 90%
Ward, 2021 [30]	USA and Japan	Phase recognition for POEM	Deep learning (CNN + LSTM)	Pre‐clinical study	Retrospective study	Not specified	Develop an AI system for POEM phase recognition and validate its performance with videos (*n* = 20): overall accuracy 87.6%; unweighted and prevalence‐weighted F1 scores 0.766 and 0.875
Ebigbo, 2022 [31]	Germany, Canada, and Brazil	Delineation of vessels, tissue structures, and instruments	Deep learning (DeepLabv3)	Pre‐clinical study	Retrospective study	Not specified	Develop an AI system to detect vessels, tissue and instruments during third space endoscopy. Performance measured by internal cross validation and external videos: mean IoU, Dice Score and pixel accuracy 63%, 76%, and 81%; mean vessel detection rate 85%
Jiang, 2023 [32]	Japan	Detection and localization of perforations	Deep learning (YOLOv3)	Pre‐clinical study	Retrospective study	Not specified	Develop an AI system for perforations detection and validate its performance with videos (*n* = 49): accuracy, AUC and precision of 0.881, 0.869, and 0.879

Abbreviations: ADR, adenoma detection rate; AI, artificial intelligence; AMR, adenoma miss rate; AUC, area under the curve; BLI, blue light imaging; CADe, computer‐aided detection; CADx, computer‐aided diagnosis; CBD, common bile duct; CE, capsule endoscopy; CNN, convolutional neural network; CRNN, convolutional recurrent neural network; DCNN, deep convolutional neural network; DSOC, digital single‐operator cholangioscopy; ERCP, endoscopic retrograde cholangiopancreatography; ESD, endoscopic submucosal dissection; EUS, endoscopic ultrasound; LSTM, long short‐term memory; NPV, negative predictive value; PDAC, pancreatic ductal adenocarcinoma; POEM, peroral endoscopic myotomy; PPV, positive predictive value; RCT, randomized controlled trial; SBCE, small bowel capsule endoscopy; WCE, wireless capsule endoscopy; WLI, white light imaging.

## 4 Results—The Present Landscape

Current AI applications are actively enhancing endoscopy training across various domains. The present landscape is discussed here.

### 4.1 Luminal Endoscopy

#### 4.1.1 Computer‐Aided Detection

**FIGURE 2:**
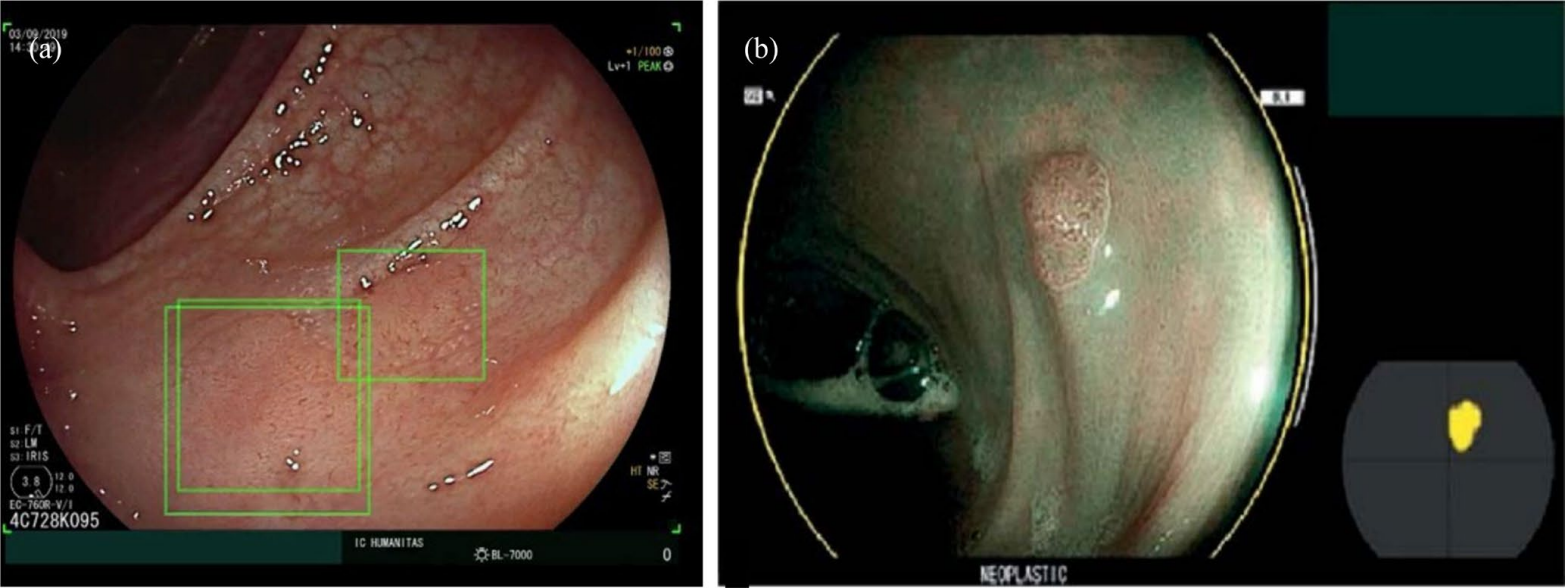
Examples of AI application in luminal endoscopy. (a) Computer‐aided detection (CADe; GI Genius, Medtronic) applied by Repici et al. [6] (b) Computer‐aided characterization (CADx; CAD‐EYE; Fujifilm) applied by Weight et al. [11]

Currently, computer‐aided detection (CADe) for colonic polyps detection is the best‐developed and well‐validated AI application in luminal endoscopy (Figure 2). Multiple randomized trials have highlighted the potential benefits of CADe, and a recent meta‐analysis [33] demonstrated that AI‐assisted colonoscopy significantly improved adenoma detection rate (ADR), regardless of location, size, and morphology. While most of these studies involved experienced endoscopists, a few of them specifically assessed its performance in less‐experienced operators, which were particularly relevant to endoscopy training and education. A European study demonstrated a 22% increase in ADR and a 21% increase in adenoma per colonoscopy (APC) among non‐expert endoscopists using CADe compared to controls [6]. Following that, our group performed a randomized trial to specifically assess the benefits of CADe in trainees. Trainees using CADe showed a 13% absolute and a 41% relative increase in ADR compared to those without CADe. Subgroup analysis revealed a stronger impact on the most junior trainees—58% relative increase in ADR among beginners and a 36% relative increase in ADR among intermediate‐level trainees [7]. Another clinical trial conducted in Japan compared the use of a combined CADe and CADx system versus standard observation by trainees during colonoscopy. The AI‐assisted group had a significantly lower adenoma miss rate (AMR) and higher scores in pathology identification or location interpretation [8]. Moreover, a Chinese study compared novice endoscopists with or without AI assistance versus experts. The performance of novice endoscopists with AI assistance was superior to those without AI, and achieved a result comparable to experts in terms of AMR and polyp miss rate. These promising and consistent results supported the potential integration of AI into colonoscopy training, especially in the early career stage to enhance the overall learning experience and quality control [9].

#### 4.1.2 Computer‐Aided Diagnosis

Moving forward from CADe, computer‐aided diagnosis (CADx) systems to distinguish neoplastic and non‐neoplastic polyps have been advocated in a number of prospective studies and meta‐analyses (Figure 2) [34]. However, their role in endoscopy training remains nebulous. A pilot study by Peng et al. demonstrated that CADx significantly improved trainees' negative predictive value for differentiating polyps' characteristics from 50.0% to 88.2% [10]. Similarly, Weight et al. reported that non‐expert endoscopists using CADx achieved similar accuracy with experts, suggesting its possible value for trainees [11]. In contrast, Rex et al. found no meaningful improvement in sensitivity and only a marginal increase in specificity when evaluating a CADx system for general endoscopists, across all experience levels [12]. Taken these into account, the heterogenous results pose uncertainties regarding the role of CADx in endoscopic training and require further validations.

#### 4.1.3 Computer‐Aided Quality Assessment

Apart from the well‐known CADe and CADx, other novel AI‐driven technologies in luminal endoscopy have the potential to enhance endoscopy training. Computer‐aided quality assessment (CAQ) systems [35, 36] could evaluate endoscopic performance in real time, offering trainees actionable feedback to refine procedural skills. These systems typically assess quality metrics such as blind spot recognition, withdrawal speed, effective withdrawal time, movement patterns, and mucosal fold examination quality. Additionally, an AI‐powered automated reporting system was proposed to streamline endoscopy documentation processes and improve accuracy [13]. These technological innovations illustrate the expanding role of AI in augmenting endoscopy training, decision‐making, and workflow efficiency. Yet, more clinical trials would be necessary to prove their potential benefits due to the limited existing real‐world evidence.

### 4.2 Hepatobiliary Endoscopy

#### 4.2.1 Endoscopic Ultrasound

**FIGURE 3:**
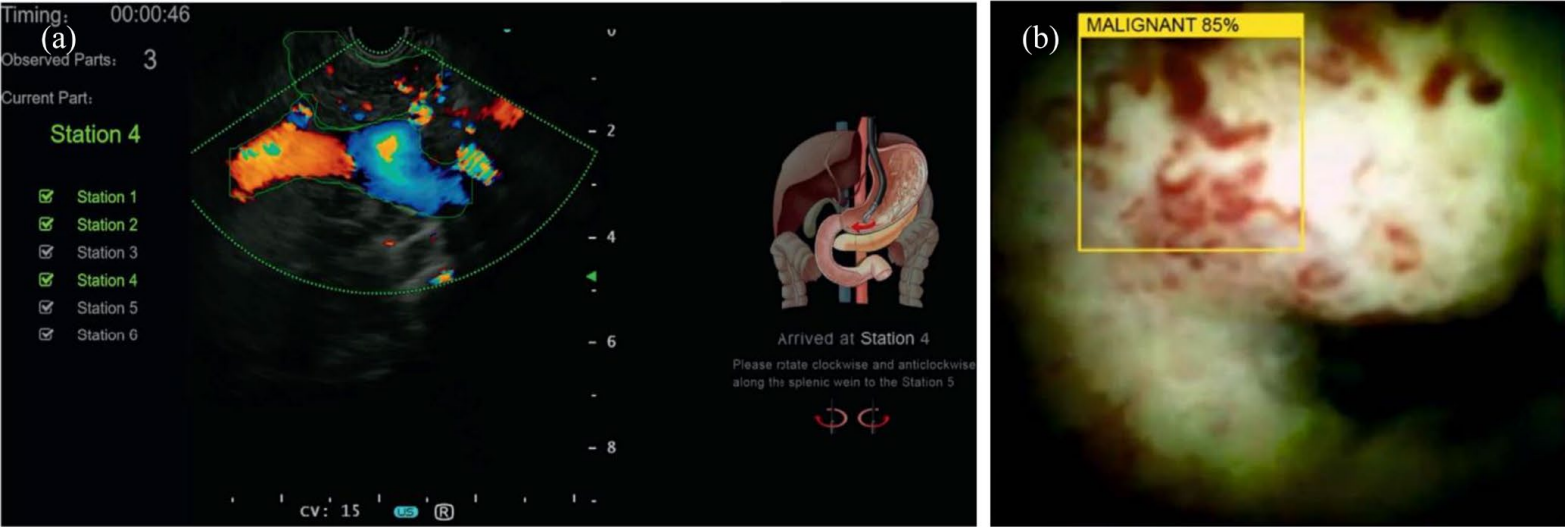
Examples of AI application in hepatobiliary endoscopy. (a) Station recognition in endoscopic ultrasound (EUS) (EUS‐based convolutional neural network model developed by Robles‐Medranda et al. [14]) (b) Detection of neoplasm during cholangioscopy (digital single‐operator cholangioscopy‐based convolutional neural network model developed by Robles‐Medranda et al. [22]).

AI applications in endoscopic ultrasound (EUS) training are advancing in three key domains, namely structure/station recognition, lesion diagnosis, and quality control (Figure 3). Robles‐Medranda et al. developed an AI system capable of identifying normal anatomical structures in both pre‐recorded videos and real‐time EUS. By improving image interpretation and highlighting critical landmarks, this tool may flatten the learning curve and maintain procedural consistency [14]. Furthermore, a deep learning‐based EUS training system could achieve 90.0% accuracy in station classification with Dice coefficients of 0.77 (blood vessel segmentation) and 0.813 (pancreas segmentation), matching expert‐level performance. A crossover study revealed that this model boosted trainees' station recognition accuracy from 67.2% to 78.4% [15]. With system upgrades involving bile duct annotation and station recognition, it further raised novices' time‐point accuracy from 60.8% to 76.3% [16].

For lesion diagnosis, a deep‐learning‐based system for real‐time capture and segmentation of solid pancreatic masses was introduced. In a crossover trial, trainees using the system showed improved performance metrics, with an increased intersection over union (IoU) from 0.80 to 0.87. In addition, lesion identification times decreased in the pancreatic body/tail (from 22.75 to 17.98 s) and the head/uncinate process (from 34.21 to 25.92 s) [17]. Cui et al. developed a multi‐modal AI system integrating clinical data and EUS images to distinguish pancreatic carcinoma from benign lesions. In the study, AI assistance improved novice endoscopists' diagnostic accuracy from 0.69 to 0.90. It also enhanced expert endoscopists' acceptance of the predictions from AI by offering supplementary interpretability [18]. Moreover, a deep‐learning radiomics (DLR) model was proposed to identify pancreatic ductal adenocarcinoma (PDAC), achieving high accuracy, sensitivity, and specificity. Junior endoscopists using the DLR system matched or exceeded the performance of senior endoscopists, supporting its potential role as an educational aid [19].

Regarding quality control, an AI‐based automatic EUS image reporting system was developed, which outperformed manual assessments in both accuracy and completeness for standard biliopancreatic stations. This innovation could serve as a potential tool for training standardization [20].

#### 4.2.2 Endoscopic Retrograde Cholangiopancreatography

AI in endoscopic retrograde cholangiopancreatography (ERCP) and cholangioscopy training is still emerging, with most efforts primarily focusing on procedural difficulty prediction and lesion detection (Figure 3). A deep‐learning‐based system was developed to evaluate the complexity of common bile duct (CBD) stone extraction during ERCP. The system automatically assessed stone size, CBD diameter, and technical difficulty, which may offer actionable plans to trainees in selecting therapeutic strategies and minimizing adverse events [21]. Moreover, an AI model for real‐time detection of neoplastic lesions during digital single‐operator cholangioscopy was superior to non‐expert endoscopists in diagnostic accuracy. This model may potentially boost trainees' diagnostic confidence, reduce missed lesions, and accelerate skill acquisition [22]. Nevertheless, the number of prospective clinical trials in the field of hepatobiliary endoscopy was still limited, providing promising yet premature evidence for implementation.

### 4.3 Capsule Endoscopy

**FIGURE 4:**
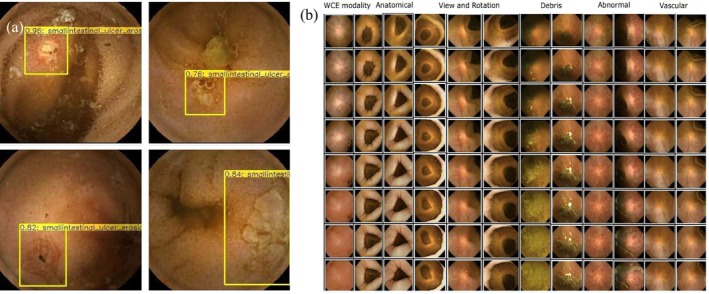
Examples of AI application in capsule endoscopy. (a) Detection of erosions and ulcerations (Single Shot MultiBox Detector developed by Aoki et al. [24]) (b) Atlas creation (StyleGAN2‐based generation of wireless capsule endoscopy images developed by Vats et al. [27]).

AI is enhancing the training of capsule endoscopy (CE) by shortening the reading time, improving lesion detection, and creating an atlas with diverse pathologies for education (Figure 4). Postgate et al. evaluated a computer‐based CE training system among 28 trainees (14 medical students and 14 gastroenterology trainees). Participants reviewed video clips of normal anatomy, incidental findings, and true pathologies, then completed multiple‐choice questions with feedback. After training, the lesion recognition scores improved significantly in gastroenterology trainees (49.5%–62.1%) and medical students (29.5%–46.7%) [23]. Subsequently, a deep learning‐based screening tool for small‐bowel CE (SBCE) was developed and tested in a crossover study. AI assistance significantly reduced trainees' reading time, though the lesion detection rate for erosions or ulcerations remained unchanged. Notably, the subgroup analysis showed that trainees, regardless of any AI use, missed larger lesions (> 5 mm) detected by experts, implying a persistent gap in CE training [24]. Subsequently, another deep learning‐based system for gastric and small‐bowel lesion detection during CE was developed. With the support of AI, the video reading time was reduced, and more lesions were identified. In particular, junior endoscopists' diagnostic metrics and accuracies were improved [25]. In a validation study for automatic SBCE diagnosis, junior endoscopists using AI achieved higher overall accuracy and sensitivity while maintaining similar specificity compared to unaided assessments, reinforcing its potential role in CE education [26]. Last but not least, a style‐based generative adversarial network was developed to generate a high‐quality synthetic atlas of highly realistic and diverse small bowel pathologies from anonymized patient data. This offers a scalable resource for CE training and education [27].

### 4.4 Advanced Therapeutic Endoscopy

**FIGURE 5:**
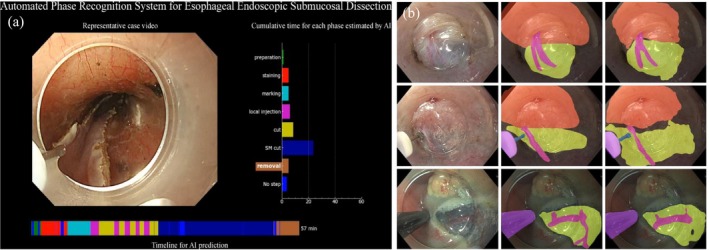
Examples of AI application in therapeutic endoscopy. (a) Phase recognition during endoscopic submucosal dissection (phase recognition‐based deep neural network model developed by Furube et al. [29]) (b) Detection and delineation of vessels, tissue structures, and instruments (artificial intelligence clinical decision support solution developed by Ebigbo et al. [31]).

Procedural phase recognition and real‐time identification of critical features (e.g., blood vessels, dissection planes, and perforations) are essential elements for advanced therapeutic endoscopy training (Figure 5). Cao et al. developed a deep learning‐based surgical workflow recognition system for endoscopic submucosal dissection (ESD). The platform includes an online scoring system to evaluate procedural proficiency and smoothness, providing trainees with targeted expert feedback on techniques [28]. Furube et al. created an AI phase‐recognition system for esophageal ESD with a 90% accuracy, facilitating rapid phase review for novice endoscopists [29]. Additionally, Ward et al. extended this concept to peroral endoscopic myotomy (POEM), achieving a phase‐identification accuracy of 87.6% with the potential for training standardization [30].

To enhance the safety and precision during these complex procedures, Ebigbo et al. designed an AI model based on still images to outline vessels and tissue structures. The algorithm achieved an 85% mean vessel detection rate with a false‐positive rate of 0.75/min, potentially reducing intra‐procedural complication risk and accelerating skill acquisition [31]. Furthermore, the same group validated this algorithm through video review by 19 endoscopists with different experience levels. The vessel detection rate increased from 56.4% to 72.4%, while the vessel detection time was reduced from 6.7 to 5.2 s [37]. On the other hand, an AI system was trained for perforation detection and localization and achieved an accuracy and AUC of 0.881 and 0.869 respectively, which may assist intraoperative decision‐making by inexperienced endoscopists [32]. The existing level of evidence supports further development in this field, but further testing of these systems in clinical trial settings will be mandatory for future applications.

## 5 Discussion

### 5.1 The Future—Foreseeing the Next Generation Endoscopy Training With AI

The development of AI in GI endoscopy is thriving, but significant gaps remain. Future AI systems must evolve beyond straightforward tasks like lesion identification to encompass a comprehensive and personalized training solution. In addition, AI may also reshape education for trainers.

#### 5.1.1 End‐To‐End Application—From Pre‐Procedure to Post‐Procedure

##### 5.1.1.1 Pre‐Procedure

AI can facilitate pre‐procedure learning through advanced simulation training [38]. For instance, augmented reality (AR) and virtual reality (VR) platforms powered by AI could create immersive, high‐fidelity, and risk‐free environments for trainees to practice. These simulations could replicate diverse pathological conditions, which may be difficult to encounter in real‐world settings. Additionally, AI could assist in pre‐procedure planning by analyzing patient history, imaging results, and risk factors to recommend personalized strategies, such as the mode of sedation [39]. This would not only enhance trainees' mental preparedness but also improve patient safety. Furthermore, using large language models in medical education has shown favorable results and deserves further exploration in cognitive skills training [40, 41].

##### 5.1.1.2 Intra‐Procedure

With the rapid development of CADe and CADx, future AI may support real‐time decision‐making regarding the necessity and modality of endoscopic resection—a common challenge to trainees [42]. Besides, AI also shows the potential to provide intraprocedural guidance and corrective feedback on endoscopic navigation, thereby expediting the learning curve for fine motor control [43, 44]. In therapeutic endoscopy, AI could guide trainees through complex interventions, such as hemostasis or submucosal dissection [45]. Such real‐time assistance is particularly valuable for beginners with a huge need for mentorship.

##### 5.1.1.3 Post‐Procedure

After endoscopic procedures, AI systems could provide detailed evaluations of trainees' performance by analyzing metrics such as procedure time, lesion detection rates, navigation efficiency, and photo documentation quality [46, 47, 48]. This may serve as an objective assessment tool for trainees' competence and facilitate trainers in providing constructive feedback.

#### 5.1.2 Personalized Training

Under the traditional apprenticeship‐based training model, trainees' competency is often determined by training time or volume rather than their actual performance. Mastery learning is an emerging concept that tailors training according to trainees' skills, as they must attain a pre‐determined level of proficiency before progressing to more advanced stages [49]. AI may assist mastery learning by converting subjective milestones to data‐driven and scalable proficiency assessments (e.g., performance scores, learning curves, or time‐to‐proficiency). For advanced trainees and trained endoscopists, AI‐powered performance analytics may identify areas for improvement and recommend targeted training modules.

#### 5.1.3 Train‐The‐Trainer Integration

Teaching attributes and endoscopy attributes are essential qualities of an effective endoscopy trainer [50], but formal training for trainers is often lacking. Trainers may not recognize their own teaching gaps, and not all of them are equally skilled in coaching nuanced endoscopic techniques. For the teaching aspect, AI‐based natural language processors may monitor trainer–trainee interaction with feedback, such as clarity of instruction and completeness of debriefing. In addition, AI may also elevate trainers' endoscopy aspect through targeted upskilling and reskilling exercise.

### 5.2 Potential Harms of AI Integration

#### 5.2.1 Deskilling

Integrating AI into endoscopic training risks deskilling, where trainees may lose essential basic competencies due to their dependence on technology [51]. One of the major concerns is the decline in diagnostic proficiency. AI systems that automatically detect and characterize lesions may lead to underdeveloped visual recognition skills. Endoscopists‐in‐training might become dependent on AI to identify subtle mucosal changes, potentially compromising their ability to interpret endoscopic findings independently without AI. Besides, studies have shown that AI can limit the visual tracking of endoscopists [52], a crucial skill and quality metric for a thorough examination of the gastrointestinal tract [53, 54]. While mitigation strategies have been proposed to enhance active visual tracking [55], the broader concern remains: transition from traditional teaching to AI‐driven training may reduce learning by exploration, as trainees follow algorithmic guidance rather than engaging in the iterative discovery process. This shift could diminish critical thinking, as trainees might prioritize AI suggestions over foundational knowledge, such as understanding pathophysiology and clinical relevance [56]. Over time, this could diminish endoscopists' ability to integrate clinical context into their judgment and decisions.

#### 5.2.2 Over‐Reliance

Another significant drawback is the over‐reliance on AI, which can lead to unintended consequences. Automation bias is a well‐documented phenomenon where users uncritically accept AI‐generated outputs, even when they are incorrect [57]. In the context of endoscopic training, this could mean that trainees fail to challenge AI's mistakes, such as missed lesions or false‐positive signals. Additionally, AI systems often prioritize common findings and filter out “atypical” lesions that they were never trained on. This could reduce trainees' awareness and exposure to rarer pathologies, limiting their readiness for real‐life encounters with uncommon and complex findings [58]. A further disadvantage is the reduced preparedness for emergencies in the event of system breakdowns, since automation may degrade situational awareness [59]. Trainees accustomed to AI assistance may struggle to perform independently when the technology is unavailable. These points highlight the need for a balanced approach to AI integration into training curriculums to maintain endoscopists' ability to function effectively with or without AI support.

#### 5.2.3 Ethics

Adopting AI in endoscopic training also raises ethical concerns, particularly on liability, informed consent, and the dehumanization of patient care. Liability issues are overwhelming as the legal framework for AI use in healthcare remains ambiguous. In case of errors due to AI‐generated advice, it is unclear whether the responsibility lies with the AI developer, the training institution, or the individual clinician. This legal ambiguity complicates risk management and could deter the adoption of AI in training programs. Moreover, informed consent presents another ethical dilemma, as patients may not fully understand the role of AI during their procedures. As AI algorithms are well‐known to contain “black boxes,” their decision‐making process is notoriously difficult to explain to laymen. Ensuring transparency and obtaining consent in such scenarios is crucial but challenging [60]. Finally, the dehumanization of patient care is a growing concern [61]. Most AI systems focus on quality metrics, such as polyp detection rates, which may overshadow the importance of patient‐centered clinical care. Trainees might become overly reliant on AI outputs and neglect the human aspects of patient care, such as empathy and contextual understanding. This could lead to a generation of clinicians who are technically proficient but less adept at building rapport with patients or addressing their psychological needs. Overcoming these ethical challenges requires robust frameworks that prioritize patient rights, data security, and the preservation of humanistic values in medical training.

#### 5.2.4 Practical Limitations

AI systems may not be equitable across training centers due to the significant investment and technical support incurred. Besides, challenges related to AIs' compatibility with existing infrastructure can impede their integration. Additionally, gaps in educator literacy and familiarity with AI tools may restrict their utilization even when available. These practical hurdles may inadvertently widen disparities and compromise the standardization of endoscopy training, creating a “two‐tier” situation—trainees in well‐equipped centers can benefit from cutting‐edge AI tools, while others would lack such exposure.

### 5.3 The Ways Forward

While utilization of AI in endoscopy training is expected, its integration must be carefully managed to address ethical, psychological, and societal concerns. As AI's performance is highly dependent on the endoscopist's knowledge [62], the importance of traditional teaching must not be undermined. A holistic framework is vital to ensure that AI complements, rather than replaces, conventional training.

First, AI should be introduced into training programs in a structured and phased manner. This involves setting clear objectives for AI use, such as improving procedural skills or standardizing training outcomes. Institutions should also establish guidelines for AI adoption, ensuring that its use aligns with their respective educational goals and ethical standards. A hybrid training approach, combining AI with mentorship from experienced clinicians, is crucial for balancing technological and human elements. While AI can provide objective feedback and real‐time guidance, mentors offer invaluable insights into clinical judgment, patient communication, and gatekeeping for automation bias [63]. This human–AI synergy has shown promise in surgical skill instruction and is poised to yield similar results in endoscopy education [64].

While over‐reliance on AI systems may result in de‐skilling, AI can be tailor‐made to enhance trainees' skills by offering personalized feedback based on one's strengths and weaknesses. Future AI should incorporate objective learning outcomes for progress tracking. In addition, practical barriers must be overcome before the generalized adoption of AI. Although AI implementation was shown to be cost‐saving in screening colonoscopy [65], its cost‐effectiveness in endoscopy training remains uncertain and warrants further investigation. Experienced endoscopists should be equipped with knowledge of AI. Ultimately, universal adoption of AI may reduce variability and harmonize endoscopy training by delivering consistent and objective feedback—an advantage particularly relevant in units with limited access to expertise and mentorship.

To address concerns about algorithmic bias, all AI systems should be validated by experts in diverse clinical settings to ensure their accuracy and generalizability before incorporation into training curricula. On the other hand, an AI system trained predominantly on data from a particular demographic group might underperform in another group. Therefore, institutions must conduct regular audits of the AI systems to evaluate their performance continuously and ensure that they are equitable and dependable.

Navigating ethical concerns requires a multifaceted approach. To address the liability ambiguities, governing bodies should establish shared accountability frameworks that clearly define responsibilities among AI developers, clinicians, and healthcare organizations. For instance, developers could be held accountable for algorithmic errors, while clinicians retain responsibility for final decisions, ensuring human oversight remains central. [66] Mandatory documentation of AI use in procedural reports, like when and how AI guidance was followed, would enhance traceability and clarify liability in case of untoward outcomes. To tackle the “black‐box” critique, developers should prioritize explainable AI (XAI) methods that make algorithmic decisions interpretable to clinicians and patients [67, 68]. Regulatory bodies could further support transparency by mandating the disclosure of training data composition and the performance limitations of AI systems. Last but not least, engaging patients in informed consent processes explicitly, by outlining how their data is used and the AI's role in their clinical care, respects autonomy and fosters trust in the use of AI.

## 6 Conclusion

AI is reshaping the landscape of GI endoscopy. Although AI can address many challenges that we are facing in endoscopic education, much is still unknown about how it should be incorporated. Undoubtedly, its implementation must be supported by a holistic framework that prioritizes ethical considerations, mitigates risks, and preserves the human elements of medicine. By adopting a hybrid and stepwise approach, ensuring quality of AI models, and focusing on personalized development, we can benefit from the power of AI to enhance endoscopy training efficiency and ultimately improve patient care.

## Author Contributions

J.C.L.H., Z.Q., and L.H.S.L. contributed equally to the literature review, data analysis, and manuscript writing. H.‐C.Y. and P.W.Y.C. were responsible for the critical review of the manuscript. All authors approved the final manuscript.

## Conflicts of Interest

L.H.S.L. has research collaborations with Olympus Co. Ltd. and GenieBiome Ltd., and served as an advisory board member for AstraZeneca and GenieBiome Ltd., and served as a lecture speaker for Olympus Co. Ltd., Boston Scientific Co. Ltd., Pfizer Inc., and GenieBiome Ltd. H.‐C.Y. has research collaborations with Olympus Co. Ltd., and served as a lecture speaker for Olympus Co. Ltd., Cornerstone Robotics Ltd., Medtronic Hong Kong Medical Ltd., and Creo Medical Ltd. P.W.Y.C. has a research collaboration with Olympus Co. Ltd. and Boston Scientific; and served as an advisor for EndoVision and EndoMaster; and as a lecture speaker for Olympus. The other authors declare no conflicts of interest.

## References

[1] C. Le Berre , W. J. Sandborn , S. Aridhi , et al., “Application of Artificial Intelligence to Gastroenterology and Hepatology,” Gastroenterology 158 (2020): 76–94.e2. 31593701 10.1053/j.gastro.2019.08.058

[2] V. Kaul , S. Enslin , and S. A. Gross , “History of Artificial Intelligence in Medicine,” Gastrointestinal Endoscopy 92 (2020): 807–812. 32565184 10.1016/j.gie.2020.06.040

[3] V. Wadhwa , M. Alagappan , A. Gonzalez , et al., “Physician Sentiment Toward Artificial Intelligence (Ai) in Colonoscopic Practice: A Survey of Us Gastroenterologists,” Endoscopy International Open 8 (2020): E1379–E1384. 33015341 10.1055/a-1223-1926 PMC7508643

[4] W. W. Goh , K. Y. Chia , M. F. Cheung , et al., “Risk Perception, Acceptance, and Trust of Using Ai in Gastroenterology Practice in the Asia‐Pacific Region: Web‐Based Survey Study,” JMIR Artificial Intelligence 3 (2024): e50525. 38875591 10.2196/50525 PMC11041476

[5] P. J. Schulz , M. O. Lwin , K. M. Kee , W. W. B. Goh , T. Y. T. Lam , and J. J. Y. Sung , “Modeling the Influence of Attitudes, Trust, and Beliefs on Endoscopists' Acceptance of Artificial Intelligence Applications in Medical Practice,” Frontiers in Public Health 11 (2023): 1301563. 38089040 10.3389/fpubh.2023.1301563 PMC10715310

[6] A. Repici , M. Spadaccini , G. Antonelli , et al., “Artificial Intelligence and Colonoscopy Experience: Lessons From Two Randomised Trials,” Gut 71 (2022): 757–765. 34187845 10.1136/gutjnl-2021-324471

[7] L. H. S. Lau , J. C. L. Ho , J. C. T. Lai , et al., “Effect of Real‐Time Computer‐Aided Polyp Detection System (Endo‐Aid) on Adenoma Detection in Endoscopists‐In‐Training: A Randomized Trial,” Clinical Gastroenterology and Hepatology 22 (2024): 630–641.e4. 37918685 10.1016/j.cgh.2023.10.019

[8] D. Yamaguchi , R. Shimoda , K. Miyahara , et al., “Impact of an Artificial Intelligence‐Aided Endoscopic Diagnosis System on Improving Endoscopy Quality for Trainees in Colonoscopy: Prospective, Randomized, Multicenter Study,” Digestive Endoscopy 36 (2024): 40–48. 37079002 10.1111/den.14573 PMC12136242

[9] L. Yao , X. Li , Z. Wu , et al., “Effect of Artificial Intelligence on Novice‐Performed Colonoscopy: A Multicenter Randomized Controlled Tandem Study,” Gastrointestinal Endoscopy 99 (2024): 91–99.e9. 37536635 10.1016/j.gie.2023.07.044

[10] C. Peng , C. X. Tian , Y. Mu , et al., “Hyperspectral Imaging Facilitating Resect‐And‐Discard Strategy Through Artificial Intelligence‐Assisted Diagnosis of Colorectal Polyps: A Pilot Study,” Cancer Medicine 13 (2024): e70195. 39320133 10.1002/cam4.70195 PMC11423483

[11] J. Weigt , A. Repici , G. Antonelli , et al., “Performance of a New Integrated Computer‐Assisted System (Cade/Cadx) for Detection and Characterization of Colorectal Neoplasia,” Endoscopy 54 (2022): 180–184. 33494106 10.1055/a-1372-0419

[12] D. K. Rex , I. Bhavsar‐Burke , D. Buckles , et al., “Artificial Intelligence for Real‐Time Prediction of the Histology of Colorectal Polyps by General Endoscopists,” Annals of Internal Medicine 177 (2024): 911–918. 38768450 10.7326/M24-0086

[13] L. Zhang , Z. Lu , L. Yao , et al., “Effect of a Deep Learning‐Based Automatic Upper Gi Endoscopic Reporting System: A Randomized Crossover Study (With Video),” Gastrointestinal Endoscopy 98 (2023): 181–190.e10. 36849056 10.1016/j.gie.2023.02.025

[14] C. Robles‐Medranda , J. Baquerizo‐Burgos , M. Puga‐Tejada , et al., “Development of Convolutional Neural Network Models That Recognize Normal Anatomic Structures During Real‐Time Radial‐Array and Linear‐Array Eus (With Videos),” Gastrointestinal Endoscopy 99 (2024): 271–279.e2. 37827432 10.1016/j.gie.2023.10.028

[15] J. Zhang , L. Zhu , L. Yao , et al., “Deep Learning‐Based Pancreas Segmentation and Station Recognition System in Eus: Development and Validation of a Useful Training Tool (With Video),” Gastrointestinal Endoscopy 92 (2020): 874–885.e3. 32387499 10.1016/j.gie.2020.04.071

[16] L. Yao , J. Zhang , J. Liu , et al., “A Deep Learning‐Based System for Bile Duct Annotation and Station Recognition in Linear Endoscopic Ultrasound,” eBioMedicine 65 (2021): 103238. 33639404 10.1016/j.ebiom.2021.103238 PMC7921468

[17] A. Tang , P. Gong , N. Fang , et al., “Endoscopic Ultrasound Diagnosis System Based on Deep Learning in Images Capture and Segmentation Training of Solid Pancreatic Masses,” Medical Physics 50 (2023): 4197–4205. 36965116 10.1002/mp.16390

[18] H. Cui , Y. Zhao , S. Xiong , et al., “Diagnosing Solid Lesions in the Pancreas With Multimodal Artificial Intelligence: A Randomized Crossover Trial,” JAMA Network Open 7 (2024): e2422454. 39028670 10.1001/jamanetworkopen.2024.22454 PMC11259905

[19] J. Gu , J. Pan , J. Hu , et al., “Prospective Assessment of Pancreatic Ductal Adenocarcinoma Diagnosis From Endoscopic Ultrasonography Images With the Assistance of Deep Learning,” Cancer 129 (2023): 2214–2223. 36999572 10.1002/cncr.34772

[20] X. Li , L. Yao , H. Wu , et al., “A Deep Learning‐Based, Real‐Time Image Report System for Linear Eus,” Gastrointestinal Endoscopy 101 (2024): 1166–1173. 39427992 10.1016/j.gie.2024.10.030

[21] L. Huang , X. Lu , X. Huang , et al., “Intelligent Difficulty Scoring and Assistance System for Endoscopic Extraction of Common Bile Duct Stones Based on Deep Learning: Multicenter Study,” Endoscopy 53 (2021): 491–498. 32838430 10.1055/a-1244-5698

[22] C. Robles‐Medranda , J. Baquerizo‐Burgos , J. Alcivar‐Vasquez , et al., “Artificial Intelligence for Diagnosing Neoplasia on Digital Cholangioscopy: Development and Multicenter Validation of a Convolutional Neural Network Model,” Endoscopy 55 (2023): 719–727. 36781156 10.1055/a-2034-3803 PMC10374349

[23] A. Postgate , A. Haycock , S. Thomas‐Gibson , et al., “Computer‐Aided Learning in Capsule Endoscopy Leads to Improvement in Lesion Recognition Ability,” Gastrointestinal Endoscopy 70 (2009): 310–316. 19386301 10.1016/j.gie.2008.11.043

[24] T. Aoki , A. Yamada , K. Aoyama , et al., “Clinical Usefulness of a Deep Learning‐Based System as the First Screening on Small‐Bowel Capsule Endoscopy Reading,” Digestive Endoscopy 32 (2020): 585–591. 31441972 10.1111/den.13517

[25] X. Xie , Y. F. Xiao , H. Yang , et al., “A New Artificial Intelligence System for Both Stomach and Small‐Bowel Capsule Endoscopy,” Gastrointestinal Endoscopy 100 (2024): 878.e1–878.e14. 10.1016/j.gie.2024.06.004 38851456

[26] Z. Ding , H. Shi , H. Zhang , et al., “Artificial Intelligence‐Based Diagnosis of Abnormalities in Small‐Bowel Capsule Endoscopy,” Endoscopy 55 (2023): 44–51. 35931065 10.1055/a-1881-4209

[27] A. Vats , M. Pedersen , A. Mohammed , and Ø. Hovde , “Evaluating Clinical Diversity and Plausibility of Synthetic Capsule Endoscopic Images,” Scientific Reports 13 (2023): 10857. 37407635 10.1038/s41598-023-36883-x PMC10322862

[28] J. Cao , H. C. Yip , Y. Chen , et al., “Intelligent Surgical Workflow Recognition for Endoscopic Submucosal Dissection With Real‐Time Animal Study,” Nature Communications 14 (2023): 6676. 10.1038/s41467-023-42451-8 PMC10590425 37865629

[29] T. Furube , M. Takeuchi , H. Kawakubo , et al., “Automated Artificial Intelligence‐Based Phase‐Recognition System for Esophageal Endoscopic Submucosal Dissection (With Video),” Gastrointestinal Endoscopy 99 (2024): 830–838. 38185182 10.1016/j.gie.2023.12.037

[30] T. M. Ward , D. A. Hashimoto , Y. Ban , et al., “Automated Operative Phase Identification in Peroral Endoscopic Myotomy,” Surgical Endoscopy 35 (2021): 4008–4015. 32720177 10.1007/s00464-020-07833-9 PMC7854950

[31] A. Ebigbo , R. Mendel , M. W. Scheppach , et al., “Vessel and Tissue Recognition During Third‐Space Endoscopy Using a Deep Learning Algorithm,” Gut 71 (2022): 2388–2390. 36109151 10.1136/gutjnl-2021-326470 PMC9664130

[32] K. Jiang , H. Itoh , M. Oda , et al., “Gaussian Affinity and Giou‐Based Loss for Perforation Detection and Localization From Colonoscopy Videos,” International Journal of Computer Assisted Radiology and Surgery 18 (2023): 795–805. 36913126 10.1007/s11548-022-02821-x

[33] J. Makar , J. Abdelmalak , D. Con , B. Hafeez , and M. Garg , “Use of Artificial Intelligence Improves Colonoscopy Performance in Adenoma Detection: A Systematic Review and Meta‐Analysis,” Gastrointestinal Endoscopy 101 (2025): 68–81.e8. 39216648 10.1016/j.gie.2024.08.033

[34] E. Young , L. Edwards , and R. Singh , “The Role of Artificial Intelligence in Colorectal Cancer Screening: Lesion Detection and Lesion Characterization,” Cancers (Basel) 15 (2023): 5126. 37958301 10.3390/cancers15215126 PMC10647850

[35] K. M. Cold , A. Vamadevan , A. S. Vilmann , M. B. S. Svendsen , L. Konge , and F. Bjerrum , “Computer‐Aided Quality Assessment of Endoscopist Competence During Colonoscopy: A Systematic Review,” Gastrointestinal Endoscopy 100 (2024): 167–176.e1. 38580134 10.1016/j.gie.2024.04.004

[36] S. M. Chan , D. Chan , H. C. Yip , et al., “Artificial Intelligence‐Assisted Esophagogastroduodenoscopy Improves Procedure Quality for Endoscopists in Early Stages of Training,” Endoscopy International Open 13 (2025): a25476645. 40309064 10.1055/a-2547-6645 PMC12042994

[37] M. W. Scheppach , R. Mendel , A. Muzalyova , et al., “Use of Artificial Intelligence in Submucosal Vessel Detection During Third‐Space Endoscopy,” Endoscopy 57 (2025): 760–766. 39909396 10.1055/a-2534-1164

[38] M. Finocchiaro , P. Cortegoso Valdivia , A. Hernansanz , et al., “Training Simulators for Gastrointestinal Endoscopy: Current and Future Perspectives,” Cancers (Basel) 13 (2021): 1427. 33804773 10.3390/cancers13061427 PMC8004017

[39] S. Syed , M. Syed , F. Prior , et al., “Machine Learning Approach to Optimize Sedation Use in Endoscopic Procedures,” Studies in Health Technology and Informatics 281 (2021): 183–187. 34042730 10.3233/SHTI210145 PMC9016977

[40] A. Skryd and K. Lawrence , “Chatgpt as a Tool for Medical Education and Clinical Decision‐Making on the Wards: Case Study,” JMIR Formative Research 8 (2024): e51346. 38717811 10.2196/51346 PMC11112466

[41] H. Mondal , J. K. K. Karri , S. Ramasubramanian , S. Mondal , A. Juhi , and P. Gupta , “A Qualitative Survey on Perception of Medical Students on the Use of Large Language Models for Educational Purposes,” Advances in Physiology Education 49 (2025): 27–36. 39447120 10.1152/advan.00088.2024

[42] Y. Kamitani , K. Nonaka , and H. Isomoto , “Current Status and Future Perspectives of Artificial Intelligence in Colonoscopy,” Journal of Clinical Medicine 11 (2022): 2923. 35629049 10.3390/jcm11102923 PMC9143862

[43] C. M. Hsu , C. C. Hsu , Z. M. Hsu , T. H. Chen , and T. Kuo , “Intraprocedure Artificial Intelligence Alert System for Colonoscopy Examination,” Sensors (Basel) 23 (2023): 1211. 36772251 10.3390/s23031211 PMC9921893

[44] S. Thakkar , N. M. Carleton , B. Rao , and A. Syed , “Use of Artificial Intelligence‐Based Analytics From Live Colonoscopies to Optimize the Quality of the Colonoscopy Examination in Real Time: Proof of Concept,” Gastroenterology 158 (2020): 1219–1221.e2. 31945357 10.1053/j.gastro.2019.12.035 PMC7103545

[45] M. W. Scheppach , H. C. Yip , Y. Chen , et al., “Feasibility of Real‐Time Artificial Intelligence‐Assisted Anatomical Structure Recognition During Endoscopic Submucosal Dissection,” Endosc Int Open 13 (2025): a26158008. 40611836 10.1055/a-2615-8008 PMC12223949

[46] Y. Y. Chang , H. H. Yen , P. C. Li , et al., “Upper Endoscopy Photodocumentation Quality Evaluation With Novel Deep Learning System,” Digestive Endoscopy 34 (2022): 994–1001. 34716944 10.1111/den.14179

[47] V. Rajan , H. Srinath , C. Y. S. Bong , A. Cichowski , C. J. Young , and P. J. Hewett , “Software Analysis of Colonoscopy Videos Enhances Teaching and Quality Metrics,” Cureus 14 (2022): e23039. 35464512 10.7759/cureus.23039 PMC9001872

[48] M. Wittbrodt , M. Klug , M. Etemadi , A. Yang , J. E. Pandolfino , and R. N. Keswani , “Assessment of Colonoscopy Skill Using Machine Learning to Measure Quality: Proof‐Of‐Concept and Initial Validation,” Endoscopy International Open 12 (2024): E849–E853. 38966321 10.1055/a-2333-8138 PMC11221895

[49] H. Maulahela , N. G. Annisa , T. Konstantin , A. F. Syam , and R. Soetikno , “Simulation‐Based Mastery Learning in Gastrointestinal Endoscopy Training,” World Journal of Gastrointestinal Endoscopy 14 (2022): 512–523. 36186944 10.4253/wjge.v14.i9.512 PMC9516469

[50] C. Wells , “The Characteristics of an Excellent Endoscopy Trainer,” Frontline Gastroenterology 1 (2010): 13–18. 28839536 10.1136/fg.2009.000372 PMC5517162

[51] R. Sparrow and J. Hatherley , “The Promise and Perils of Ai in Medicine,” International Journal of Chinese and Comparative Philosophy of Medicine 17 (2019): 79–109.

[52] J. Troya , D. Fitting , M. Brand , et al., “The Influence of Computer‐Aided Polyp Detection Systems on Reaction Time for Polyp Detection and Eye Gaze,” Endoscopy 54 (2022): 1009–1014. 35158384 10.1055/a-1770-7353 PMC9500006

[53] M. Lami , H. Singh , J. H. Dilley , et al., “Gaze Patterns Hold Key to Unlocking Successful Search Strategies and Increasing Polyp Detection Rate in Colonoscopy,” Endoscopy 50 (2018): 701–707. 29415286 10.1055/s-0044-101026

[54] C. Almansa , M. W. Shahid , M. G. Heckman , S. Preissler , and M. B. Wallace , “Association Between Visual Gaze Patterns and Adenoma Detection Rate During Colonoscopy: A Preliminary Investigation,” American Journal of Gastroenterology 106 (2011): 1070–1074. 21326224 10.1038/ajg.2011.26

[55] A. Sivananthan , J. Ahmed , A. Kogkas , G. Mylonas , A. Darzi , and N. Patel , “Eye Tracking Technology in Endoscopy: Looking to the Future,” Digestive Endoscopy 35 (2023): 314–322. 36281784 10.1111/den.14461 PMC12136275

[56] F. Cabitza , R. Rasoini , and G. F. Gensini , “Unintended Consequences of Machine Learning in Medicine,” JAMA 318 (2017): 517–518. 28727867 10.1001/jama.2017.7797

[57] L. Introzzi , J. Zonca , F. Cabitza , P. Cherubini , and C. Reverberi , “Enhancing Human‐Ai Collaboration: The Case of Colonoscopy,” Digestive and Liver Disease 56 (2024): 1131–1139. 37940501 10.1016/j.dld.2023.10.018

[58] R. Challen , J. Denny , M. Pitt , L. Gompels , T. Edwards , and K. Tsaneva‐Atanasova , “Artificial Intelligence, Bias and Clinical Safety,” BMJ Quality and Safety 28 (2019): 231–237. 10.1136/bmjqs-2018-008370 PMC6560460 30636200

[59] M. R. Endsley , “From Here to Autonomy,” Human Factors 59 (2017): 5–27. 28146676 10.1177/0018720816681350

[60] T. Ploug and S. Holm , “The Four Dimensions of Contestable Ai Diagnostics ‐ a Patient‐Centric Approach to Explainable Ai,” Artificial Intelligence in Medicine 107 (2020): 101901. 32828448 10.1016/j.artmed.2020.101901

[61] P. Formosa , W. Rogers , Y. Griep , S. Bankins , and D. Richards , “Medical Ai and Human Dignity: Contrasting Perceptions of Human and Artificially Intelligent (Ai) Decision Making in Diagnostic and Medical Resource Allocation Contexts,” Computers in Human Behavior 133 (2022): 107296.

[62] J. Lee , W. S. Cho , B. S. Kim , et al., “Impact of User's Background Knowledge and Polyp Characteristics in Colonoscopy With Computer‐Aided Detection,” Gut Liver 18 (2024): 857–866. 39054913 10.5009/gnl240068 PMC11391145

[63] K. Goddard , A. Roudsari , and J. C. Wyatt , “Automation Bias: A Systematic Review of Frequency, Effect Mediators, and Mitigators,” Journal of the American Medical Informatics Association 19 (2012): 121–127. 21685142 10.1136/amiajnl-2011-000089 PMC3240751

[64] B. Giglio , A. Albeloushi , A. K. Alhaj , et al., “Artificial Intelligence‐Augmented Human Instruction and Surgical Simulation Performance: A Randomized Clinical Trial,” JAMA Surgery 160 (2025): 993–1003. 40768205 10.1001/jamasurg.2025.2564 PMC12329680

[65] M. Areia , Y. Mori , L. Correale , et al., “Cost‐Effectiveness of Artificial Intelligence for Screening Colonoscopy: A Modelling Study,” Lancet Digital Health 4 (2022): e436–e444. 35430151 10.1016/S2589-7500(22)00042-5

[66] W. N. Price, 2nd , S. Gerke , and I. G. Cohen , “Potential Liability for Physicians Using Artificial Intelligence,” JAMA 322 (2019): 1765–1766. 31584609 10.1001/jama.2019.15064

[67] E. J. Topol , “High‐Performance Medicine: The Convergence of Human and Artificial Intelligence,” Nature Medicine 25 (2019): 44–56. 10.1038/s41591-018-0300-7 30617339

[68] C. Rudin , “Stop Explaining Black Box Machine Learning Models for High Stakes Decisions and Use Interpretable Models Instead,” Nature Machine Intelligence 1 (2019): 206–215. 10.1038/s42256-019-0048-x PMC9122117 35603010

